# Reduction and fixation of proximal humeral fracture with severe medial instability using a small locking plate

**DOI:** 10.1186/s12893-021-01388-9

**Published:** 2021-10-31

**Authors:** Yuelei Zhang, Lifu Wan, Lecheng Zhang, Chao Yan, Gang Wang

**Affiliations:** Department of Orthopedics, First Affiliated Hospital of Anhui Medical University, 218 Jixi Road, 230000 Hefei, China

**Keywords:** Anteromedial locking plate, Humeral fracture, Humeral calcar, Medial support, PHILOS

## Abstract

**Background:**

Currently, the reduction and support of comminuted medial cortex of humeral fracture remains a challenge, Therefore, a novel reduction and fixation technique that employs an anteromedial small locking plate was explored in this study, and its viability and the associated complications were assessed.

**Methods:**

Fifteen cases of proximal humeral fractures with medial instability (five cases were classified as three-part and ten as four-part by Neer classification) were treated by the proposed reduction technique using an anteromedial small locking plate. Subsequently, the radiological and clinical outcomes were evaluated over an average follow-up period of 18.53 months.

**Results:**

The average operation time was 108 min (range, 70–130 min), and the mean fracture union time in all patients was 12.13 weeks (range, 8–16 weeks). Complications such as infection and neurovascular injury were not observed. Postoperative X-ray showed avascular necrosis and screw penetration in one patient, while screw penetration, varus malunion, or significant reduction loss was not found in the other cases. The mean Constant score was 79.8 (range, 68–92) during the final visit.

**Conclusions:**

The use of an anteromedial small locking plate improved the reduction efficiency, reconstructed the medial support, and alleviated the occurrence of complications in proximal humeral fractures with medial instability.

## Background

Proximal humeral fractures account for 4–5% of all fractures and predominantly occur in elderly patients. Neer has stated that most proximal humeral fractures with slight or no displacement could be treated conservatively [1]. However, recent studies have found that up to 64% of proximal humeral fractures are displaced [2]. Since conservative therapy is associated with several complications, surgical treatment of displaced proximal humeral fracture is advocated. The latter can provide anatomical reduction and stable fixation, allowing early functional exercise with improved function [3].

Although various methods such as applying traction on the arm and suture, elevator, and joystick techniques [4, 5] have been proposed, the overall anatomical or acceptable fracture reduction remains low, which results in a high complication rate. This is especially true for unstable and displaced proximal humeral fractures involving the anatomical neck or with disruption of the medial hinge [6, 7]. Furthermore, anatomical fracture reduction and correct alignment of the medial cortices are the two most important prognostic factors in terms of secondary displacement, and the disrupted medial hinge should be reconstructed prior to the application of additional reduction maneuvers [8]. However, reconstruction of the medial hinge could only be achieved through indirect manipulations, considering the complexity of the neighboring structures. Therefore, fractures with medial instability remain technically difficult to tackle.

In this study, we have reported the radiological and functional outcomes of our novel technique for managing these fractures. The findings asserted that an anteromedial locking plate effectively improves the reduction quality and helps in reconstructing the medial hinge in proximal humeral fractures with medial instability.

## Methods

### Patients

This research was approved by the Institutional Review Board of the authors’ affiliated institutions. From January 2018 to July 2020, 72 patients with operated proximal humeral fractures were admitted to our study. All patients signed written informed consent forms, including the requirement for internal fixation, and the statement of double plates when necessary. The inclusion criterion was the presence of comminuted metaphyseal proximal humeral fractures. The exclusion criteria were pathologic fractures, open fractures, fractures with neurovascular injury, fractures of > 2 weeks, and those combined with other fractures involving the ipsilateral upper limb. Finally, 15 proximal humeral fractures with severe medial instability were reduced and fixed with the help of an anteromedial locking plate. Six men and nine women were included in the study, the average age of the patients was 61.53 years (range, 32–76 years), and all of them were right-handers. The left arm was affected in four patients, and the right arm was affected in 11 patients. All patients had an explicit history of trauma, including four cases of bicycle injuries, 10 cases of walking injuries, and one case of falling from height. All fractures were closed without combined injuries to the same limb. The average time from injury to surgery was 6.6 days (range, 2–14 days). Before surgery, anterior–posterior (AP) and lateral radiographs of the shoulder were taken, and additional computed tomography and 3-dimensional reconstruction images were recommended in all patients to understand the fracture patterns and the degree of displacement. The fractures were classified using the Neer system; five cases were three-part fractures, and ten were four-part fractures.

### Surgical technique

**Fig. 1:**
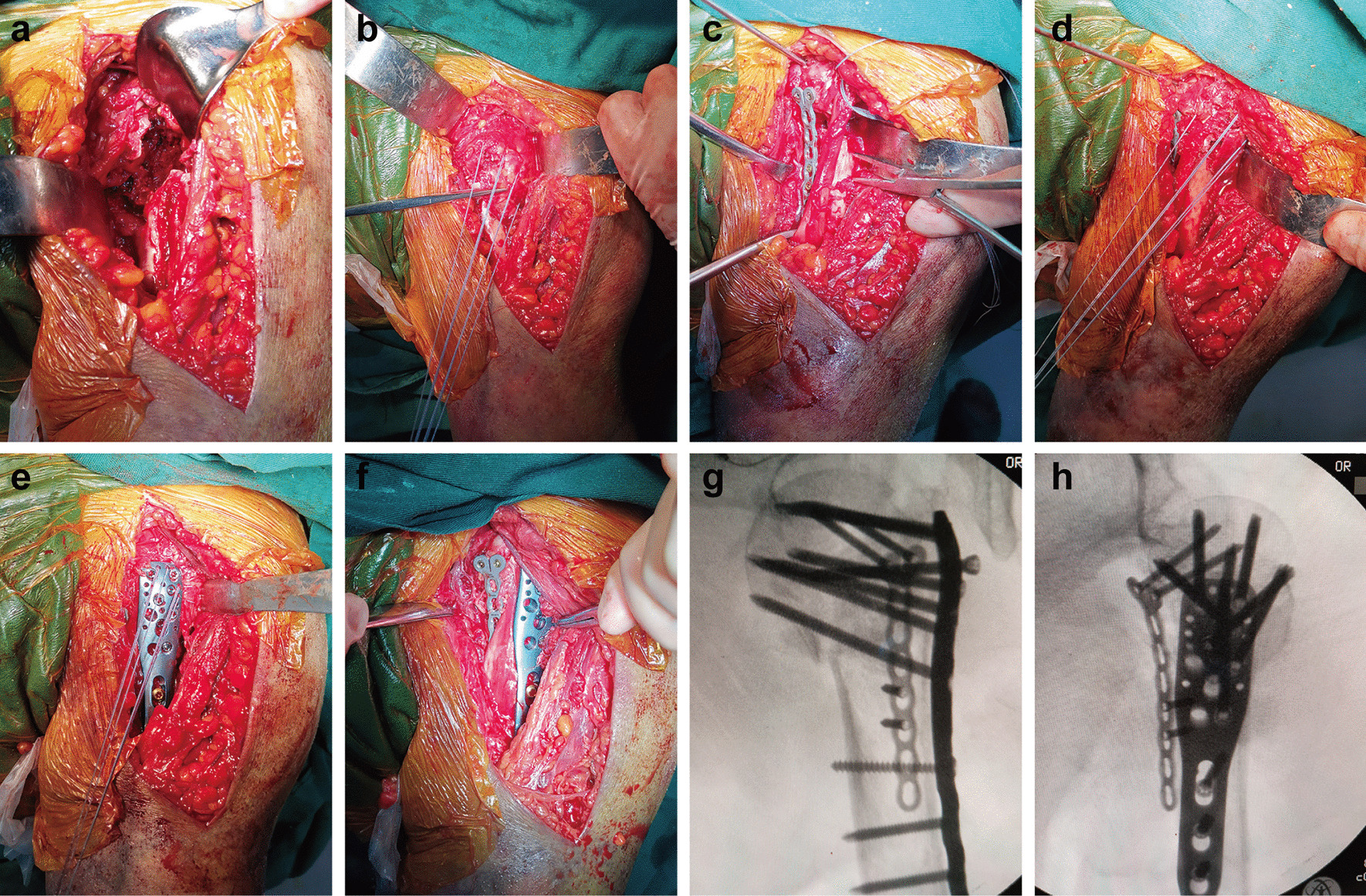
Surgical procedure involved in the reduction technique in one humeral proximal fracture with medial instability. **a** Images obtained during operation revealed comminution of the lateral cortex. **b** The suture technique was used to restore the alignment of the fractures. **c** A T-shaped 2.7-mm locking plate was placed at the medial side of LHBT, with the T-shaped head fixed on the lesser tuberosity and the distal end fixed on the shaft. **d** By using the anteromedial locking plate, the lateral cortex was easily reconstructed with the suture technique and a joystick. **e**, **f** PHILOS was placed lateral to LHBT, and the traction sutures were passed through the suture holes in order to reduce the tuberosities prior to locking screw insertion and finally tied. **g**, **h** The AP and lateral radiographs of the shoulder joint were captured and attention was paid to the use of calcar screws to further strengthen the medial support

All the surgeries were performed by the same group of surgeons. After administering general anesthesia, each patient was placed on a radiolucent table in the beach-chair position. The entire upper extremity was prepared to allow unrestricted arm traction and rotation during the operation. A deltopectoral approach was used to expose the proximal humerus; subsequently, the cephalic vein was exposed and taken medially. The deltopectoral interval was developed by finger dissection, and a Hoffmann hook was inserted beneath the deltoid muscle. After identifying the long head of the biceps brachii tendon (LHBT) in the bicipital groove, the fracture lines were separated slightly using a periosteal elevator. The coracoacromial ligament was partly cut if necessary, and the comminution of the fracture was observed by rotating the distal humerus (Fig. 1a). Later, the rotator cuff was explored, but injury was not observed in these patients. Three pairs of high-strength, non-absorbable sutures were inserted into the tendon to fix and reduce the fragments. A superior suture was used for the supraspinatus tendon, an anterior suture for the subscapularis tendon, and a posterior suture for the infraspinatus tendon as previously described [5] (Fig. 1b). Then, the alignment of the fractures was restored by pulling down the sutures along the diaphyseal axis, distraction of the humeral shaft, and levering the humeral head using the joystick technique, followed by temporary K-wire fixation and C-arm fluoroscopy. If comminuted fracture was present in the lateral cortex without any anatomical landmark or if restoring the normal alignment using existing reduction methods was difficult due to medial instability, reduction technique involving an anteromedial locking plate was selected.

First, subperiosteal dissection was performed in the medial side of the intertubercular sulcus to expose the lesser tuberosity, the anteromedial fracture line, and the cortex distal part of the fracture. The attachment of pectoralis major was stripped for 2–3 cm and repaired by suturing to the plate or by drilling the humeral cortical bone after fracture reconstruction. If necessary, the subscapularis tendon was partially released from the lesser tuberosity. The anatomical mark is usually visible in the anteromedial fracture line. According to the residual anatomical mark or the recovery of the humeral head-shaft angle (HSA), the continuity of the anterior medial cortex was restored and fixed by using the K-wire. During the restoration, techniques such as traction of the upper arm, suture technique, and joysticks were used. Subsequently, a T-shaped 2.7-mm locking plate was placed in the medial side of LHBT. The plate was pre-bent to match the anteromedial curvature of the proximal humerus, with the T-shaped head fixed on the lesser tuberosity and the distal end fixed on the shaft. The small locking plate was approximately parallel to the shaft of the humerus crossing the anteromedial fracture line. Two and three locking screws were inserted into the proximal and distal ends of the plate, respectively, to restore and support the anteromedial cortex (Fig. 1c, d).

If several bone defects existed, β-tricalcium phosphate bioceramics were implanted to maintain the normal HSA. Then, proximal humeral internal locking system (PHILOS, Synthes, Switzerland) was placed lateral to LHBT. A non-locking, bicortical screw placed in the oblong hole allowed compression of the plate against the humeral shaft and minor adjustments in the final plate positioning (Fig. 1e, f). Fluoroscopy was used to evaluate the position of the plate and avoid impingement. The traction sutures were pulled through the suture holes to reduce the tuberosities prior to the insertion of the locking screw and were finally tied. It was ensured that the screws were 5 mm lower than the articular surface as far as possible and did not pass through the cartilage surface of the humeral head, which was monitored using the AP, lateral, and axillary radiographs of the shoulder joint (Fig. 1g, h). At the same time, attention was paid to the use of calcar screws to further strengthen the medial support.

### Postoperative management

After the surgery, the patients were placed in a sling and were immediately encouraged to do pendulum exercises. The scope of passive activities was increased gradually over a period of 2–6 weeks. Active stretching, flexion, abduction, adduction, and pronation training were started 6 weeks after the operation. However, weightlifting with the injured arm was forbidden until bone union was observed.

Regular follow-up was done at 4, 8, and 12 weeks as well as at 6 and 12 months after the surgery until the shoulder functioning reached a plateau. Postoperative complications (infection, neurovascular injury, and flap necrosis), time of bone union, and radiation complications (loss of reduction, varus malunion, screw penetration, and avascular osteonecrosis) were evaluated. Bone union was defined as bony bridging on both views, and was confirmed by clinical examination [9]. The functional outcomes were evaluated at the final follow-up examination according to the Constant score. Loss of reduction was defined as HSA reduction > 10° and varus malunion as HSA reduction < 120° according to the methodology of Wang [10].

## Results

**Fig. 2:**
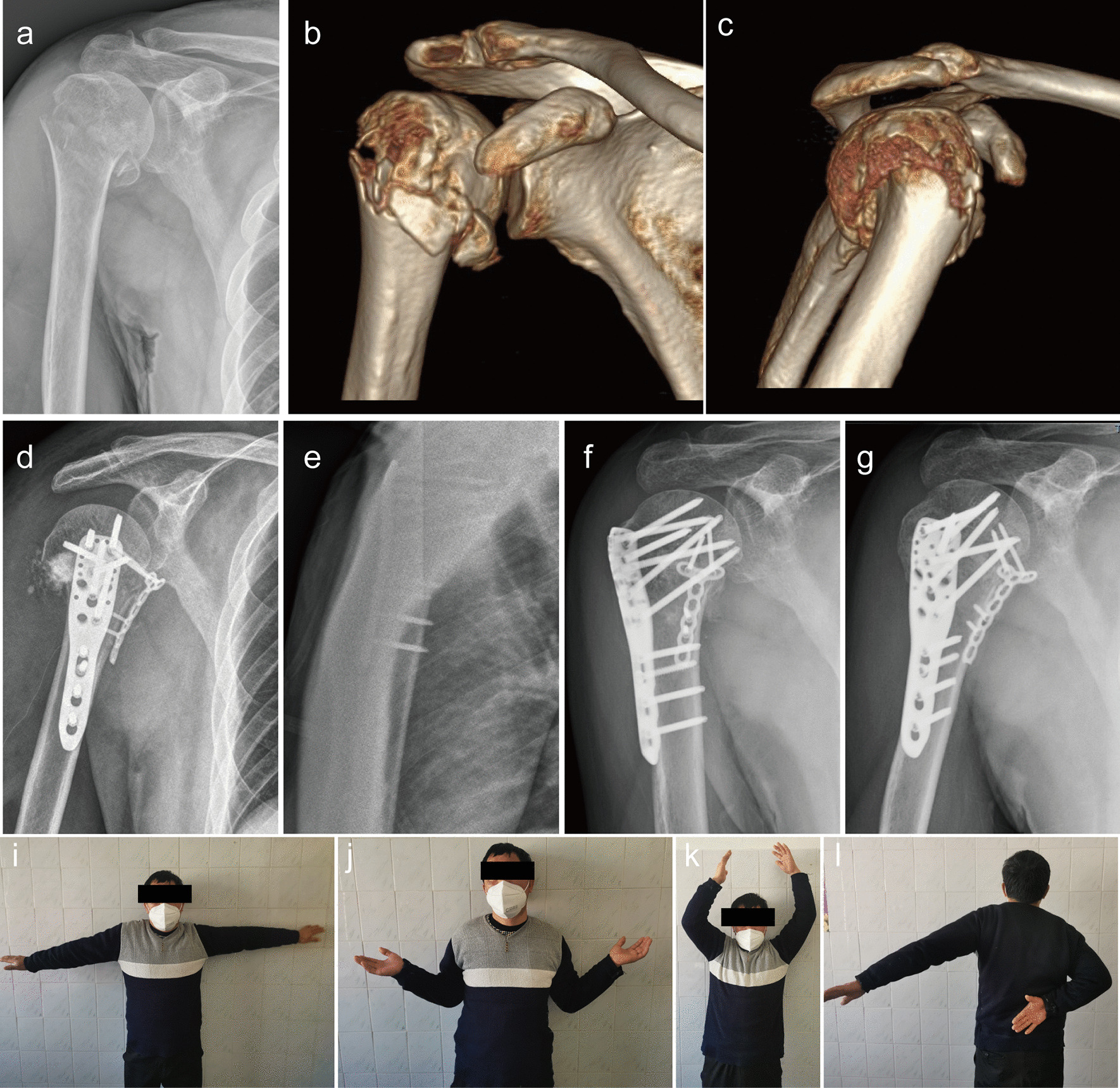
A 43-year-old patient with Neer 4-part proximal humeral fracture treated with the reduction technique. The AP (**a**) radiograph demonstrating displaced proximal humeral fracture, with the lateral radiograph unperformed due to severe pain. The CT reconstruction image (**b**, **c**) displaying a Neer 4-part fracture without any medial support. This patient was treated with PHILOS combined with a small anteromedial locking plate (**d**, **e**). X-ray images (**f**, **g**) at 11-weeks post-operation showing a bony union of the fracture. Clinical function was satisfactory at 1 year after the surgery (**i**–**l**)

**Fig. 3:**
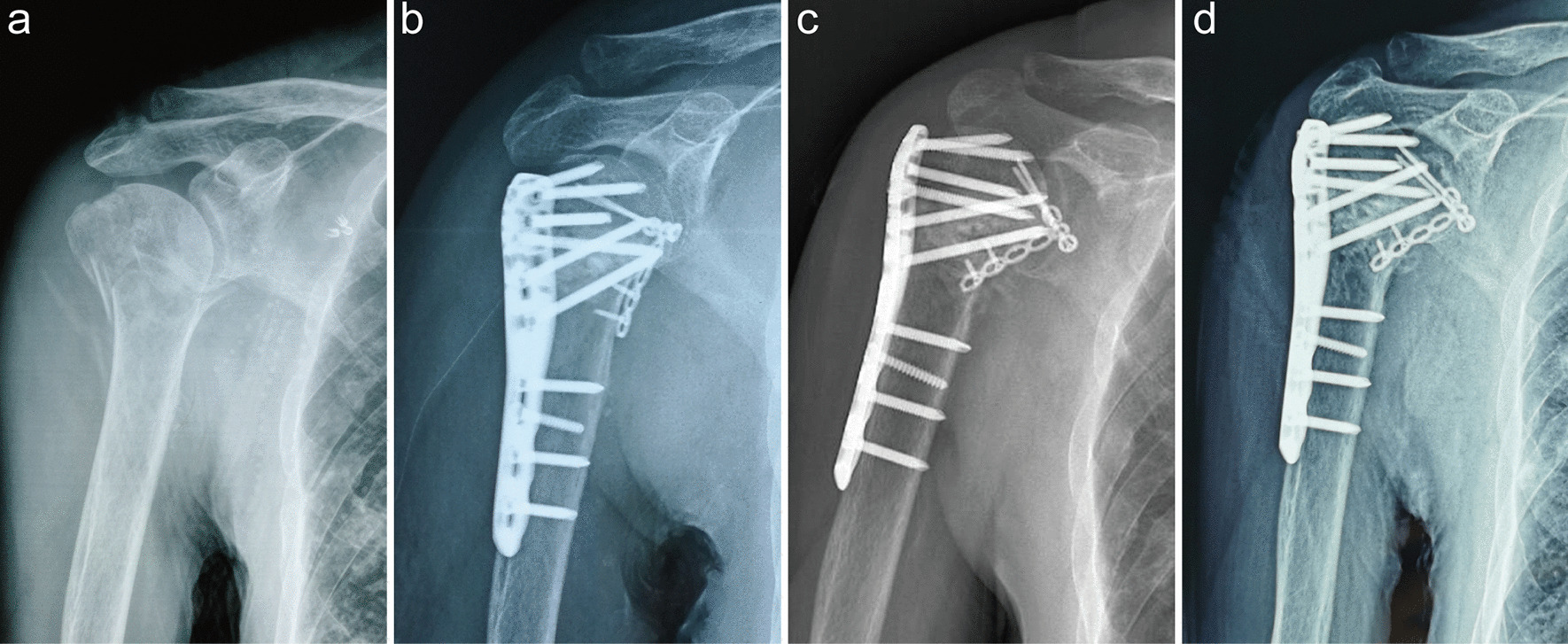
A 69-year-old patient with avascular necrosis of the humeral head. The AP (**a**) radiograph showing a Neer 3-part proximal humeral fracture. This patient was treated with PHILOS combined with a small anteromedial locking plate (**b**). The X-ray images obtained at 11 weeks after the surgery showing bony union of the fracture (**c**). Avascular necrosis of the humeral head and screw penetration were observed 14 months after the surgery (**d**)

The average operative time was 108 min (range, 70–130 min). All patients were followed up for an average period of 18.53 months (range, 9–26 months). Fracture union was achieved in all patients at a mean of 12.13 weeks (range, 8–16 weeks). No delayed unions or non-unions were observed (Fig. 2). No major complications, including infection and neurovascular injury, were noted. According to the post-surgical X-ray images, avascular necrosis and screw penetration were seen in one patient who had poor shoulder function at 14 months after the operation (Fig. 3). However, screw penetration, osteonecrosis, varus malunion or significant reduction loss was not found in the other patients. The mean Constant score was 79.8 (range, 68–92), and the shoulder functioning was satisfactory except in one patient during the final visit (Table 1).

**Table 1:** Overall characteristics and results of the 15 patients

Case no.	Sex	Age	Cause of injury	Infected shoulder	Neer classification	Union time (weeks)	Follow-up (months)	Constant score	Complication
1	M	63	WI	R	IV	12	14	82	/
2	M	43	BI	R	IV	11	16	90	/
3	F	57	WI	R	IV	13	15	79	/
4	F	65	WI	R	III	16	16	73	/
5	F	74	WI	L	IV	14	16	75	/
6	M	46	FFH	R	III	12	18	90	/
7	M	65	WI	R	IV	13	18	83	/
8	F	69	WI	R	III	11	18	68	Avascular necrosis screw penetration
9	F	55	BI	R	IV	12	23	84	/
10	F	72	WI	L	IV	16	26	72	/
11	F	66	WI	R	IV	14	26	84	/
12	M	32	BI	L	III	8	26	92	/
13	F	71	BI	R	IV	12	24	70	/
14	F	76	WI	L	IV	10	13	73	/
15	M	69	WI	R	III	8	9	82	/

*BI *bicycle injury, *WI w*alking injury, *FFH *falling from height

## Discussion

The treatment of displaced and unstable proximal humeral fractures remains challenging. The missing restoration of the medial cortical support has been identified to be an independent risk factor for fixation failure in such fractures. Hence, some investigators have advocated that medial reconstruction and buttress at the metaphysis are the most important prognostic factors for the use of locked-plate fixation for the proximal humerus[11, 12]. However, in complex proximal humeral fractures with metaphyseal comminution, the greater tuberosity is often displaced owing to the supraspinatus tendon and the lateral cortex is often comminuted; hence, there is no anatomical landmark for reduction. However, the anteromedial cortex medial to the bicipital groove and the calcar are not highly comminuted because of superior bone quality and can serve as anatomical landmarks, providing a reference for the anatomical reduction of the fracture. Owing to the complexity of the medial restoration, anatomical reduction and reliable fixation of the anteromedial cortex is a key concern in these complex fractures. If proximal humeral fractures cannot be effectively reduced through the lateral window, the remaining anteromedial anatomical landmarks can be used to achieve anatomical reduction. Subsequently, an anteromedial locking plate can be applied to stabilize the fixation. Owing to the high bone strength, the anteromedial locking plate could provide fine medial stability, which is not only conducive to the overall reduction of the proximal humeral fracture and the fixation of the lateral locking plate but also enhances the mechanical strength of the calcar area. At the same time, when combined with the use of bone substitutes and calcar screws, it can provide a stable medial support.

The proximal humeral internal locking system, which is contoured to the anatomy of the lateral aspect of the proximal part of the humerus with three-dimensional distributed and angular stable screws, is currently the mainstream choice in treating proximal humeral fractures. Nonetheless, its postoperative complication rate remains high (16–36%) even with the use of calcar screws [10, 13], especially for medially comminuted proximal humeral fractures. At present, additional augmentation of the medial column has been reported to improve the outcome of the locking plate system, including bone graft, bone substitute, and steel plate fixation [1, 14], and the double-plate technique appears to offer some advantages[15]. Theopold et al. [15] placed a one-third tubular plate in the bicipital groove in an inverted fashion, providing high stability in fractures with metaphyseal comminution. However, a biceps tendon tenotomy was needed to expose the bicipital groove. He et al. [16] designed a novel medial anatomical locking plate to directly support the medial column, but the placement of the anatomical plate required extensive medial stripping. In this study, we introduced a small locking plate, which was placed in an anteromedial position without invading the bicipital groove or injuring the medial neurovascular structures. With this reduction and fixation technique, we reconstructed the medial support of the proximal humerus and fixed it with the anteromedial locking plate, especially for comminuted surgical neck fractures and anatomical neck fractures. Obvious loss of HSA or varus malunion was not found during the follow-up period.

It has previously been stated that the anterolateral branch of the anterior circumflex artery mainly provides blood supply to the head of the humerus, while the posterior circumflex artery supplies only the posterior aspect of the greater tubercle and a small posteroinferior part of the articular segment [17]. Hettrich et al. [18] adopted a new method and found that overall, the posterior circumflex artery provides 64% of the blood supply to the humeral head and provides significantly more blood supply to the superior, lateral, and inferior quadrants of the humeral head. In proximal humeral fractures, the anterior circumflex artery adheres intimately to the bone of the head and is often disrupted, whereas the posterior circumflex artery is non-adherent and remains normal in most cases [19, 20]. Therefore, perfusion derived from the posterior circumflex artery alone may be sufficient for head survival, and the initial total ischemia could proceed to partial and full revascularization of the humeral head according to Hertel’s study. In our study, the anteromedial plate was positioned medial to the bicipital groove and did not affect the anterolateral branch of the anterior circumflex artery that entered the humeral head at the lateral and superior aspects of the bicipital groove if undisrupted. Furthermore, the locking mechanism of the anteromedial plate did not exert pressure on the periosteum and did not disturb the remaining blood supply to the humeral head. The high quality of reduction and stability achieved with the use of the anteromedial plate also contributed to the occurrence of revascularization in the humeral head.

In our opinion, the main indication for this technique involving an anteromedial locking plate is to reduce proximal humeral fractures in which the severe medial comminution could not supply stable medial support and the anatomical position could not be determined via the lateral window simultaneously. These could happen in about 11–25% of unstable fractures according to the clinical results in several studies [21, 22], which is similar with our observation. In these circumstances, the anteromedial cortex may be the only visible anatomical marker to assist reduction. Even when the anteromedial cortex is too comminuted to perform reduction, we should try to restore the curvature of the anteromedial cortex and then place the anteromedial locking plate. Further indication is to reinforce the medial support of fractures with severe medial instability that could be reduced with conventional reduction methods. Nevertheless, this technique should not be used as a routine method in fractures that can be reduced easily and medial support can be regained after reduction. In this technique, 2.7-mm locking plate is recommended to avoid extensive detachment and to protect the residual blood supply to the humeral head. Non-absorbable sutures placed in the tendinous insertions and the joystick technique are advocated for the process of reduction. However, it should be noted that the placement of the anteromedial plat needs further medial stripping including partial attachment of pectoralis major and subscapularis tendon, inappropriate repairment of the attachment may lead to decreasing internal rotation force of the shoulder joint. Meanwhile, medial dissection is also at risk of neurovascular injury and dead cavity formation, disruption of the remaining perfusion to the humeral head may also happens. Therefore, careful operation is necessary.

There are several limitations to this study. First, this study involved a small sample size without comparation with other fixation constructs since this technique was chosen on the basis of intraoperative reduction, there were many influence factors including the extent of medial comminution when comparation was operated. however, when compared with other techniques intending to reconstruct the medial stability [1, 9, 15], the radiological and clinical outcomes were comparable. Second, although the anteromedial locking plate achieved satisfactory outcomes, the extent of its effectiveness remains to be unclear, further biomechanical tests will be needed.

## Conclusions

To conclude, it could be stated that when compared with other reduction techniques reported in the literature, the application of the anteromedial small locking plate can improve the reduction efficiency, reconstruct the medial support effectively, and reduce the complications related to proximal humeral fractures. However, this technique is chiefly applicable for those humeral fractures with medial comminution that have lost the stability of the medial metaphysis and cannot be reduced by conventional methods.

## Data Availability

The datasets supporting the conclusions of this article are included within the article.

## Abbreviations

PHILOS: Proximal humeral internal locking system
HSA: Head-shaft angle
LHBT: Long head of the biceps brachii tendon
